# RAGE is a Potential Cause of Onset and Progression of Nonalcoholic Fatty Liver Disease

**DOI:** 10.1155/2019/2151302

**Published:** 2019-09-18

**Authors:** Kamyar Asadipooya, Kamran B. Lankarani, Rishi Raj, Mohammadreza Kalantarhormozi

**Affiliations:** ^1^Division of Endocrinology and Molecular Medicine, Department of Medicine, University of Kentucky, Lexington, KY, USA; ^2^Health Policy Research Center, Institute of Health, Shiraz University of Medical Sciences, Shiraz, Iran; ^3^Division of Endocrinology and Molecular Medicine, Department of Medicine, University of Kentucky, Lexington, KY, USA; ^4^Endocrinology and Internal Medicine, The Persian Gulf Tropical Medicine Research Center, Bushehr University of Medical Sciences, Bushehr, Iran

## Abstract

**Objective:**

Fatty liver is a rising global health concern, significantly increasing the burden of health care cost. Nonalcoholic fatty liver disease (NAFLD) has a correlation with metabolic syndrome and its complications.

**Method:**

We reviewed the literature regarding the mechanisms of developing NAFLD through AGE-RAGE signaling.

**Results:**

NAFLD, metabolic syndrome, and production of advanced glycation end-products (AGEs) share many common risk factors and appear to be connected. AGE induces production of the receptor for AGE (RAGE). AGE-RAGE interaction contributes to fat accumulation in the liver leading to inflammation, fibrosis, insulin resistance, and other complications of the fatty liver disease. The immune system, especially macrophages, has an important defense mechanism against RAGE pathway activities.

**Conclusion:**

Soluble form of RAGE (sRAGE) has the capability to reduce inflammation by blocking the interaction of AGE with RAGE. However, sRAGE has some limitations, and the best method of usage is probably autotransplantation of transfected stem cells or monocytes, as a precursor of macrophages and Kupffer cells, with a virus that carries sRAGE to alleviate the harmful effects of AGE-RAGE signaling in the settings of fatty liver disease.

## 1. Introduction

Nonalcoholic fatty liver disease (NAFLD) refers to the presence of hepatic steatosis in absence of substantial alcohol consumption or any other underlying causes of liver disease [1, 2]. It is a global health burden and is not only the most common cause of chronic liver disease in the United States but also affects middle- and low-income countries, affecting up to one-third of the adult population [3–5].

NAFLD has a wide clinical and pathological spectrum and ranges from simple fat deposition, nonalcoholic steatohepatitis (NASH), hepatocyte necrosis, and inflammation with or without fibrosis, to the end-stage liver disease that may need liver transplantation [1, 2]. Although the pathogenesis of NAFLD is not completely elucidated, hepatic steatosis seems to be an inciting event subsequently leading to inflammation, cell injury, and distortion of the cellular structure through micro- and macrovascular mechanisms [2, 6].

Risk factors for NAFLD are almost the same as risk factors for metabolic syndrome and include central obesity, insulin resistance, dysglycemia, dyslipidemia, and hypertension [7]. Dietary factors like increased intake of refined sugars, fructose, saturated fat, and cholesterol as well as decreased intake of polyunsaturated fatty acids especially omega 3 as opposed to omega 6 has been related to NAFLD progression [7, 8]. Because of its etiopathogenic similarities with metabolic syndrome, NAFLD is also considered as the hepatic component of metabolic syndrome [9].

The majority of patients with NAFLD are asymptomatic but can present with nonspecific symptoms such as fatigue, early satiety, and malaise; however, it lacks clear causality [10]. Hepatomegaly can be seen in the advanced stage, and eventually, symptoms and signs of chronic liver disease and portal hypertension ensue [11, 12].

NAFLD is diagnosed by evidence of fat accumulation in the liver by either histologic or imaging findings [5]. The histologic threshold is fat deposition in at least 5% of hepatocytes [13]. Although transabdominal sonography is the most commonly used testing modality with a sensitivity and specificity of 85% and 95%, respectively, it requires a higher fat accumulation (Up to 20–30%) for diagnosis and has high inter- and intraobserver variation [14]. The most accurate imaging for evaluation of fat deposition is T1-weighted dual-echo magnetic resonance (MR) image and proton magnetic resonance spectroscopy [15, 16]. The gold standard for diagnosis of inflammation and fibrosis is liver biopsy [14, 17]. The fibrosis (FIB)-4 score and NAFLD fibrosis score which are based on age, serum liver enzymes, serum albumin content, platelet count, and anthropometric measures and presence of diabetes mellitus have shown promise in detecting patients with fibrosis among those suffering from simple steatosis [15, 18].

Patients with NAFLD may progress to NASH, cirrhosis, and decompensation in long term [15, 19]. It is also considered a multisystem disease affecting not only the liver but also the heart and kidney among others [20–22]. Although the progression seems to be secondary to environmental factors and exposure to advanced glycation end-products (AGEs), its possibility, as well as the rate of progression, has remained a matter of debate [2, 6].

In this review, a brief discussion regarding the pathophysiology of NAFLD and the role of AGEs in the development and progression of NAFLD into NASH, cirrhosis, and hepatocellular carcinoma has been collected. Finally, we will debate the current opportunities to prevent and/or treat NAFLD and AGE-related liver injury and propose a potential therapeutic approach.

## 2. The Process of AGE, RAGE, and sRAGE Production

Diet plays an important role in the development of NAFLD. Typical dietary pattern of NAFLD patients consists of increased consumption of saturated fatty acid-, cholesterol-, and fructose-rich products. Polyunsaturated fatty acids (PUFAs) and antioxidants have protective roles against NAFLD development [23]. On the contrary, increased intake of fructose and fat-processed foods containing saturated fatty acids are the major sources of endogenous advanced glycation end-products (AGEs). [23, 24]. Based on the correlation between high fat/carbohydrate diets with NAFLD and AGEs production and the protective roles of antioxidants and diets containing PUFA, AGE production may play a crucial role in the onset and progression of NAFLD. Furthermore, aging, type 2 diabetes mellitus, obesity, and metabolic syndrome are reported as risk factors of developing NAFLD which have association with increased AGE production [23, 25, 26].

**Figure 1:**
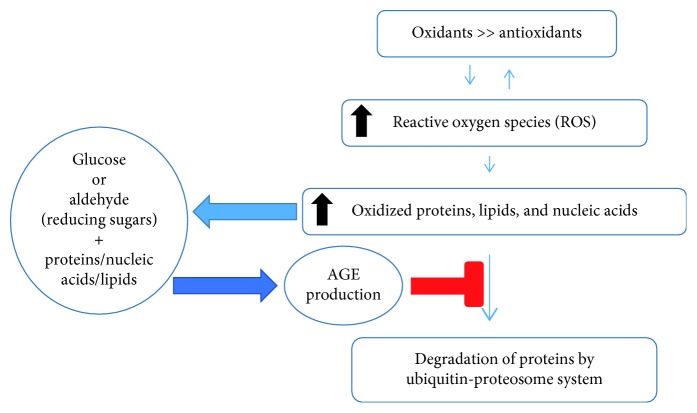
The cycle of AGE production [23].

Three different pathways participate in endogenous generation of AGEs including nonenzymatic glycation, the polyol pathway, and lipid peroxidation. The process of AGE accumulation usually takes place in the setting of hyperglycemia; however, other conditions such as aging, inflammation, renal failure, oxidative stress, consumption of high fat diet or processed food, excessive alcohol consumption, and cigarette smoking can increase endogenous or exogenous production of AGEs. The initial reaction starts with a nonenzymatic reaction between the reactive carbonyl group of reducing sugar (such as glucose, fructose, glyceraldehyde or carbonyl compounds, and acetaldehyde) and a free amino group on proteins. The reaction is reversible, produces a Schiff's base, and then undergoes spontaneous rearrangement resulting in a partially reversible Amadori product. The nonenzymatic glycation (Maillard) reaction can also happen between reducing sugar and the other macromolecules (lipids and nucleic acids); however, in human subjects with diabetes, the Maillard reaction mainly occurs between sugar, lysine, and arginine amino acid residues. Initially produced AGEs will undergo further modification, which leads to the production of heterogeneous chemical structures that can be found in different tissues and even plasma. AGE accumulation in the tissues affects proteolysis leading to increase in oxidized and damaged proteins, induction of inflammation, increase in reactive oxygen species production, and finally affecting cellular metabolic activities, which may further perpetuate AGE production (Figure 1) [23, 25].

**Figure 2:**
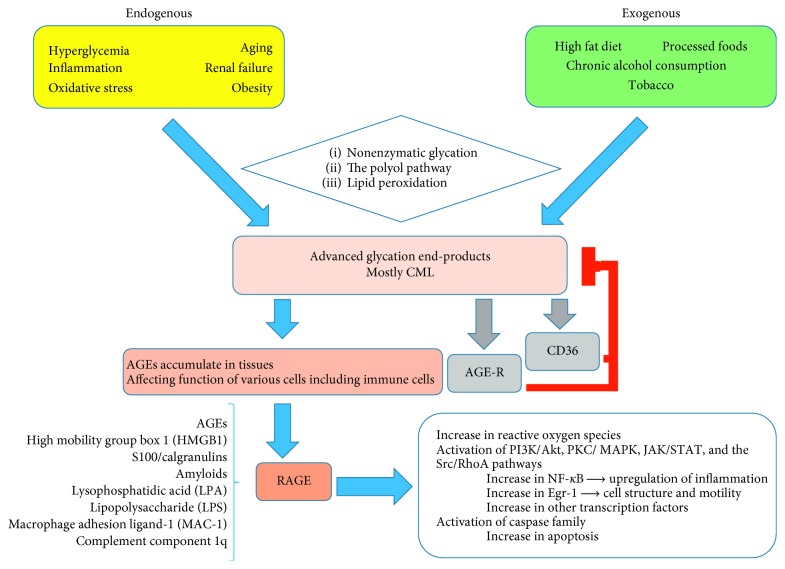
The process of AGE formation, RAGE-ligands interaction, and related consequences. CML, pentosidine, argpyrimidine, imidazolone, GOLD, MOLD, DOLD, carboxyethyl-lysine, fructose-lysine, and methylglyoxal-derived hydroimidazolones are commonly produced AGEs secondary to the reaction between carbohydrate and proteins.

AGEs not only have direct prooxidant and proinflammatory effects but also interact with variable cell surface receptors including receptor for AGEs (RAGE), scavenger receptors including class A, class B (CD36), class E (LOX-1), and class H (FEELs), and AGE receptor complexes (AGE-R). AGEs also induce production of RAGE, leading to initiation of different intrinsic signaling pathways consisting of PKC, PI3K/Akt, Src/RhoA, JAK/STAT, MAPK/ERK, and NADPH oxidase. Activation of these intrinsic signal pathways results in higher levels of NF-*κ*B, Egr-1, and/or other transcription factors which upregulate inflammation, oxidative injury affecting cellular motility, adhesion, and metabolism, ultimately causing tissue injury [24, 25, 27]. In addition, the AGE/RAGE signaling pathway can also activate apoptosis through caspase family (Figure 2) [28, 29].

**Figure 3:**
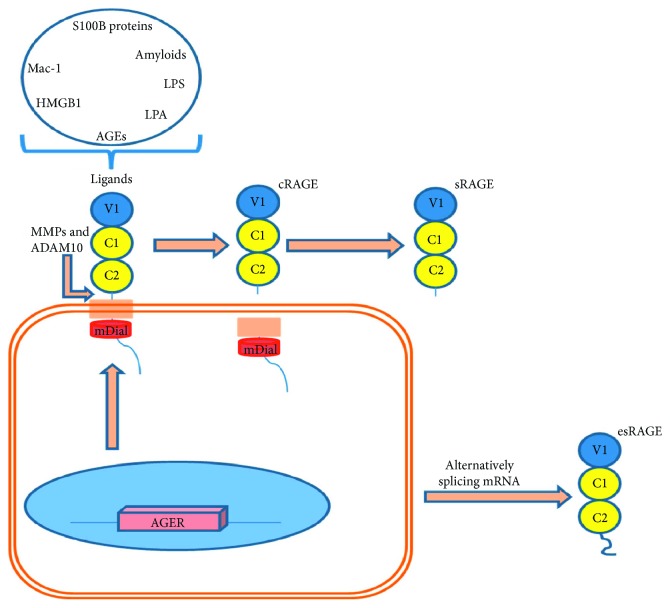
RAGE structure and the process of sRAGE formation.

RAGE is a structurally like immunoglobulin. It has extracellular portion containing V-C1-C2 domains, transmembrane domain, and intracellular domain. The V domain interacts with RAGE ligands. The cytoplasmic domain of RAGE interacts with diaphanous-1, which is required to activate the postreceptor signaling pathways [30]. DIAPH1 is a type of formin which is principally involved in cytoskeleton, interacts with microtubules, and plays a crucial role in cell organelles kinetics [31]. The interaction between DIAPH1 (mDia1) with RAGE stabilizes RAGE on cell membranes (Figure 3).

The RAGE can be expressed in different tissues and cell types including immune system cells monocyte/macrophage, lymphocyte, and neutrophil, endothelial cells, smooth muscle cells, and hepatocytes where it interacts with variable ligands [1, 23, 25]. The RAGE ligands include AGEs, HMGB1 (high mobility group box 1), LPS (lipopolysaccharides), S100/calgranulins, amyloid-β peptide (A*β*), and other forms of amyloid, lysophosphatidic acid (LPA), macrophage adhesion ligand-1 (MAC-1), and complement component 1q (C1q) (Figures 2 and 3) [23, 24, 27].

AGEs mediated pathways can be inhibited through AGE-Rs or CD36. While AGE-R detoxifies AGEs, CD36 mediates endocytosis of AGEs, leading to intracellular degradation through lysosome and is eventually cleared by the kidneys [23, 24].

RAGE signaling pathway can be potentially inhibited by different mechanisms. First, enzymatic cleavage of RAGE (cRAGE) by ADAM10 (a disintegrin and metalloproteinase domain-containing protein 10) and matrix metalloproteinase (MMPs) disrupts the external domain of the RAGE and leads to the production of soluble RAGE (sRAGE) which is a natural defense tool against AGEs. sRAGE can attach to AGEs and block AGE-RAGE interaction. sRAGE contains the extracellular portion (V-C1-C2 domains) of RAGE molecules and can be measured by the ELIZA method [25, 32]. AGEs can potentially increase production and activity of matrix metalloproteinase activity which technically reduces RAGE signaling activities and increases sRAGE production [33, 34]. Second, endogenous secretory RAGE (esRAGE) which is produced by alternatively spliced pre-mRNA of AGER gene is another form of soluble RAGE that can block AGE-RAGE interaction (Figure 3) [25, 32].

## 3. AGE-RAGE Interaction and Development of Fatty Liver

NAFLD is associated with obesity and diabetes. Liver plays a crucial role in glucose and lipid metabolism. Although the exact pathogenesis of NAFLD has not been clarified, inflammation, oxidant injury, apoptosis, and cell proliferation are the known events in NAFLD which are interconnected with glucose and lipid metabolism. The onset and development hepatic steatosis is linked with insulin resistance [35]. In addition, the complex mixture of metabolic and inflammatory signals causes the course of events from obesity to diabetes, and RAGE signaling pathway might have an important role [36].

The spectrum of NAFLD spans from isolated fat deposition (steatosis) to variable hepatocellular injury due to inflammation with or without fibrosis (NASH). Triglyceride (TG) turnover determines energy homeostasis in human. Excess accumulation of TG in hepatocytes due to increased uptake or de novo synthesis of fatty acids (FA) is an important reason for development of NAFLD [37]. In addition, increased dietary intake of cholesterol can promote fatty liver and inflammation due to mitochondrial dysfunction [35]. Excess lipid storage seems to be an initial step for hepatocellular damage. Overstorage of TG generally is associated with lipotoxicity which leads to cellular organelle dysfunction and injury, ultimately resulting in obesity and related diseases such as fatty liver, metabolic syndrome, and diabetes. Recent literature indicates that lipotoxicity mainly occurs by specific lipids such as free fatty acids (palmitic acid), diacylglycerols, cholesterol, ceramides, and lysophosphatidylcholine. Lipotoxicity causes unbalanced energy metabolism that progresses through different cellular signaling cascades including endoplasmic reticulum (ER) stress, mitochondrial dysfunction, induction of oxidative stress, and finally leading to inflammation and cellular apoptosis [37, 38].

The inflammation, cellular proliferation, and increased oxidative stress caused by AGEs lead to progression of NAFLD from simple steatosis to NASH and fibrosis [39]. On the contrary, oxidative stress and inflammation can also cause AGE production [1]. As AGEs induce RAGE signaling, the activated RAGE signaling pathway further induces oxidative stress and inflammation [23, 40]. The correlation between RAGE signaling pathway with oxidative stress and inflammation is therefore of a cyclic causation.

RAGE plays an important role in connecting innate and adaptive immune system as well as participates in the onset and maintenance of inflammation [41]. It facilitates cellular adhesion and migration of white blood cells and interacts with some of the released proinflammatory molecules such as HMBG1 and calgranulin/S100 family [42]. Mice experiments have shown that a high fat diet induces the inflammatory response leading to increase in the concentration of RAGE ligands in the liver. In addition, knocking out RAGE attenuates the effects of high fat diet on hepatic glucose production and improves insulin function in the liver [36].

The rate of cell death in the rat liver with diabetes is directly correlated to oxidative stress and expression of HMBG1 which ultimately regulates the cross-talk between apoptosis and autophagy [43]. The rate of progression of liver disease from NAFLD to fibrosis is higher among patients with elevated blood glucose levels, which can be attributed to excess AGEs and RAGE production among these groups of patients [44, 45]. This signifies the importance of AGEs having a direct involvement in progression of NAFLD through interaction with RAGE [46].

This signifies importance of AGE-RAGE signaling as an important cause of development and progression of NAFLD from simple fat deposition in hepatocytes to development of extensive liver fibrosis.

## 4. AGE-RAGE Signaling and Development of Insulin Resistance in NAFLD

Obesity and insulin resistance are the two most important contributors to the development of metabolic syndrome and NAFLD. Insulin regulates glucose and lipid metabolism. Hepatic insulin resistance is considered as an important sign or cause of NAFLD. Hepatic insulin resistance is defined as impaired suppression of hepatic glucose production through glycogenolysis and/or gluconeogenesis by insulin. It can have several different mechanisms at the molecular level including hyperglycemia with hyperinsulinemia, increased free fatty acids, and their metabolites, oxidative stress, altered profile of adipocytokines and formation of advanced glycation end-products [47].

In addition, high fat diet and endogenous fatty acid synthesis mediated by fatty acid synthase (FAS) are known to be involved in macrophage-induced pathological insulin resistance [48].

High fat diet and hyperglycemia increase formation and accumulation of AGEs. Increased AGEs and its interaction with its cellular receptor RAGE activate cascade of downstream cellular signaling and ultimately causing oxidative stress and chronic inflammation [49]. High AGE levels can also aggravate liver injury by not only the upregulation of inflammation, oxidative stress, and cytokines synthesis but also the activation of hepatic stellate cells that are involved in fat storage and fibrosis [50]. AGEs also disrupt mitochondrial energy metabolism and therefore inhibit cellular proliferation [43]. Glyoxal, which has been connected to oxidative stress and leads to AGEs formation, can cause liver mitochondrial dysfunction in a dose-dependent manner [51]. It also interferes with lipid metabolism and functions of antioxidants [52]. It seems that AGEs have a crucial role in development of hepatic insulin resistance [47, 53, 54]. Furthermore, chronic hyperglycemia and hyperinsulinemia also lead to downregulation of soluble form of RAGE (sRAGE) which again enhances the AGE-RAGE cascade leading to further insulin resistance [55].

Not only AGE-RAGE pathway leads to insulin resistance, but de novo lipid accumulation in hepatocytes also contribute to and can accentuate insulin resistance. Lipid accumulation in hepatocytes impairs intracellular signal transmission upon activation of insulin receptors leading to insulin resistance [47, 56]. The molecular events comprise progressive mitochondrial dysfunction and autophagy, increase in toxic lipids derivative intermediates (diacylglycerol -DAG- and ceramide), and decrease in total phosphatidylcholine, causing immune-mediated hepatocellular damage, ultimately resulting in hepatic insulin resistance [37, 56]. Hepatic insulin resistance also drives glucose into lipogenesis instead of glycogen synthesis leading to further hepatocyte lipid accumulation [57].

Despite strong association between AGE-RAGE signaling, insulin resistance, and NAFLD, the process of evolution from NAFLD to NASH, liver cirrhosis, and/or hepatocellular carcinoma is not only influenced by obesity and insulin resistance but also due to integrated interaction of genes, environmental factors, gut microbiome, and immune system [50]. However, presuming the correlation of the same factors contributing to hepatic insulin resistance, AGEs production, and activation of RAGE signaling, we may consider a crucial role for AGE-RAGE signaling in the development of hepatic insulin resistance due to NAFLD.

## 5. AGE-RAGE Signaling and Immune Cells, Macrophages

RAGE expression on immune cells, including macrophages, has been shown. High fat diet increases expression of RAGE ligand in the liver and adipose tissue [36]. The level of AGER mRNA is significantly higher in diabetic versus nondiabetic monocytes from the peripheral blood of human subjects [58]. AGEs have negative effects on the polarization and anti-inflammatory roles of M2 macrophages while augmenting the proinflammatory response of M1 macrophages [59].

Furthermore, sRAGE, a RAGE ligand decoy, has a promising result on reducing inflammation and prolongation of rat survival after acute liver failure and could improve hepatocytes survival after ischemic reperfusion injury [40, 60]. However, absence of RAGE on macrophages or applying sRAGE can diminish macrophage phagocytosis and clearance of apoptotic cells which is important for tissue homeostasis and modulation of inflammation [61]. On the contrary, transfection of bone marrow mesenchymal stem cells or umbilical cord-derived mesenchymal stem cells by sRAGE has more suppressive effects on inflammation and improves cell survival [60, 62]. This probably means that the transfected mesenchymal cells provide more accessible sRAGE to the area of AGE-RAGE signaling without disabling immune cells function. In other words, it seems that the cross-talk between AGE-RAGE signaling and inflammation/insulin resistance is complex, and probably initial activation of macrophages has beneficial effects, but further activation can lead to more inflammation and insulin resistance.

## 6. sRAGE and Its Correlation with NAFLD

sRAGE competes with RAGE ligands in binding to RAGE on cell membranes but does not initiate signal transduction, and thereby, it has a protective role in NAFLD. The serum levels of sRAGE in NAFLD patients are lower than the general population, and its level correlates with severity of hepatic steatosis, with higher levels of sRAGE in patients with mild disease compared to patients with moderate-to-severe hepatic steatosis [63]. Many articles have reported correlation of sRAGE and/or esRAGE with various diseases or conditions; however, the data are inconsistent. There appears to be a bidirectional correlation, which further depends on the state and/or extent of the disease process. For instance, sRAGE has a negative correlation with body mass index (BMI) and rises with antidiabetic medications (thiazolidinediones) and after bariatric surgery [64–66]. Lower levels of sRAGE are reported in atherosclerosis, hypercholesterolemia, and hypertension, whereas higher levels are found in patients with diabetes [27]. Therefore, sRAGE cannot be a reliable biomarker to assess the RAGE signaling activities as it can be elevated in either excess RAGE production or disruption. However, according to the fact that AGEs induce ADAM10 secretion, AGEs/sRAGE ratio might be helpful in the diagnosis of NAFLD patients (Table 1) [67, 78, 79].

## 7. Current Treatment, Targeting RAGE, and Future Direction

Despite significant improvement in the understanding and knowledge of etiology and pathophysiology of NAFLD, there is still no treatment modality which has been proven to be successful in the treatment of NAFLD. Life-style modifications, such as exercise and weight loss, remain the cornerstone of treatment for patients with NAFLD and NASH. Pharmacologic therapy is generally recommended for biopsy-proven NASH and high-risk situations such as liver cirrhosis and/or treating complications, such as diabetes, hypertension, or hyperlipidemia. Biguanide (metformin), peroxisome proliferator-activated receptor gamma (PPAR-γ) agonists (pioglitazone), long-acting glucagon-like peptide- (GLP-) 1 receptor agonist (liraglutide), vitamin E, lipid-lowering agents (such as statins, ezetimibe, and fibrates), antioxidants, pentoxifylline, angiotensin receptor blockers (losartan), ursodeoxycholic acid (UDCA), symbiotic or probiotics, obeticholic acid (Farnesoid X receptor agonist), and peroxisome proliferator-activated receptor alpha (PPAR-α) agonist (Elafibranor) are currently available medical therapy for treatment of NAFLD. Reduction in inflammation, fibrosis, lipid production, and hepatocellular injury by these medications leads to improvement in fatty liver disease [80]. However, these medications also play a role in reducing AGE production and/or RAGE expression, emphasizing the importance of AGE-RAGE signaling in initiation and progression of NAFLD [81–85].

As increased production of AGEs, inducing RAGE production and resulting in activation of AGE-RAGE signaling with its downstream effects at intra- and extracellular levels appear to be central in the development and progression of NAFLD, targeting AGE-RAGE signaling with AGE inhibitor (aminoguanidine), sRAGE (the ligand decoy), anti-RAGE antibody, small molecule RAGE antagonists aptamers, inhibitors of cytoplasmic domain of RAGE (ctRAGE), nanocarriers for RAGE siRNA therapy, and/or inhibitors of the HMGB1-RAGE interaction appear to be extremely promising and can potentially reduce inflammation, improve cell survival, and prevent development and/or progression of NAFLD [27, 86–89]. AGEs are structurally modified molecules, which induce inflammation after its interaction with RAGE, and therefore blocking AGEs, by means of sRAGE, and can prevent the AGE-RAGE interaction [23]. However, sRAGE does not cross the blood-brain barrier and has no significant effects on physical activity, appetite, or energy expenditure. Intraperitoneal injection of sRAGE results in significant differences in body mass between sRAGE versus vehicle-treated high fat-fed mice. sRAGE could improve insulin sensitivity and reduce inflammation. Nevertheless, sRAGE has had some limitations in blocking AGEs. It increases the levels of HMGB1 (RAGE ligand) mRNA in the liver tissue of high fat-fed mice [36]. Heterogeneity of AGEs, accessibility of sRAGE, and possible loss of beneficial effects of AGE-RAGE signaling by using sRAGE may further lessen the therapeutic effects of recombinant sRAGE [79].

Macrophages have a crucial role in initiation and maintenance of inflammation. In addition, the process of liver lipotoxicity seems to be caused by accumulation triglyceride-derived material, followed by inflammation and cellular dysfunction, which needs activity of macrophages and other immune cells [90]. Free fatty acid can increase liver macrophage accumulation which technically reiterates the process of fat accumulation as well [91]. Furthermore, the process of lobular inflammation in the liver, due to inflammatory cell infiltration and secretion of cytokines, can lead to necrosis and progression of NAFLD to NASH. However, macrophages are the resident inflammatory cells, which are involved in initiation and progression of inflammation [92].

AGE-RAGE signaling pathway plays an important role in recruiting macrophages in inflammation and inducing oxidative stress, but AGEs can increase lipid accumulation in macrophages, which disables macrophages [25, 79, 93]. In addition, AGE-RAGE signaling not only participates in insulin resistance but also seems to have some defensive role, according to the fact that knocking out RAGE leads to lower lean mass in high fat diet-fed mice [36]. Furthermore, sRAGE infusion can alleviate inflammation and macrophage phagocytosis [60, 61]. It seems AGE-RAGE signaling pathway is important for initial activation and phagocytic ability of macrophages, but further activity of the pathway may cause unregulated immune cells activity, such as macrophages, which ended up having more inflammation and tissue damage. As a result, transfecting macrophages, as a resident cell, by sRAGE may not prevent initial activation, but then after macrophages got activated, sRAGE can be secreted and probably prevent unregulated stimulation of immune cells.

Based on the capability of sRAGE in decreasing inflammation [60], the important role of macrophages, as a resident inflammatory cell, in the initiation/modulation of inflammation, reduction of macrophage phagocytosis without RAGE [61], and better suppressive effects of secreted sRAGE on inflammation and cell survival after transfection of bone marrow mesenchymal stem cells [60] or umbilical cord-derived mesenchymal stem cells [62], we may propose that watching diet and autotransplantation of transfected stem cells, or probably monocytes as a precursor of liver macrophages, by mutated lentivirus that carries sRAGE would be a reasonable approach to reduce insulin resistance and fat deposition in the liver [36, 60, 61]. Theoretically, transfected macrophages, with sRAGE, can be activated initially by being exposed to AGEs. Subsequently, transfected macrophage secretes sRAGE, blocks AGE-RAGE interaction, inhibits further activation, and reduces inflammation, fat deposition, insulin resistance, and hepatocytes injury. However, sRAGE has no effect on energy expenditure or appetite [36], may increase the levels of HMGB1 mRNA in the liver tissue of high fat-fed mice, and HMGB1 as a RAGE ligand, and has some roles in the pathogenesis of liver fibrosis through the RAGE signaling pathway [36, 67]. On the contrary, the cross-talk between oxidative stress and AGEs formation, which leads to increase in the level of AGEs, may limit the effectivity of the sRAGE. In addition, AGEs are generally complex and heterogenic in nature, which may diminish the effectivity of sRAGE in blocking AGE-RAGE interaction [23]. However, the association of certain RAGE gene polymorphisms with NASH and diabetes may indicate the dominant role of certain AGEs in the pathophysiology of NASH and diabetes, which brings up a potential hope to make a sRAGE against the dominant AGE to be able to block AGE-RAGE interaction [68, 94–96].

## 8. Conclusion

Autotransplantation of transfected stem cells or monocytes as a precursor of macrophages or Kupffer cell, with lentivirus that carries sRAGE, in addition to low carbohydrate/fat diet may give us promising results in treating NAFLD and its complications. However, we should consider limitations of sRAGE, such as accessibility, AGEs heterogeneity, and cross-talk with other RAGE ligands or oxidative stress, as a potential therapeutic approach.

## Figures and Tables

**Table 1:** Clinical studies about AGE/RAGE and NAFLD/NASH in reverse order of the publication year.

Author/Journal and year	Participants	Results	Comments
Palma-Duran et al., Metabolism, 2018 Jun. [67]	50 normoglycemic NAFLD and 58 healthy adults (age, sex, and BMI matched)	Elevated AGEs/sRAGE ratio was associated with NAFLD	AGEs (CML) higher, sRAGE levels lower, and AGEs/sRAGE ratios were higher in the NAFLD
Mehta et al., PLoS One, 2018 Jun. [68]	340 obese patients with metabolic syndrome	Certain polymorphisms of RAGE gene are indicators of NASH in obese people	Retrospective study
			Only four polymorphisms were tested
Zelber-Sagi et al., Dig Liver, Dis, 2017 May [63]	55 patients with fatty liver diagnosed by ultrasound (age 26–64)	sRAGE levels have negative correlation with liver damage	Serum sRAGE levels have negative association with fatty liver and positive correlation with fiber and vegetables consumption
Kan et al., J Clin Lab Anal, 2015 Nov. [69]	90 NBNC-HCC	AGE could discriminate NBNC-HCC from NASH	NBNC-HCC patients had higher serum levels of AGE, compared to the NASH and control subjects
	56 NASH		
	27 control subjects		
Santilli et al., Vascular Pharmacology, 2015 Sep. [70]	110 patients with FCHL, MS, and both FCHL plus MS	NAFLD patients had significantly lower plasma esRAGE irrespective of underlying metabolic abnormality (FCHL or MS)	Cross-sectional study
			Lower levels of IL-10 and adiponectin and higher CD40 ligand, endogenous thrombin potential, and oxidized LDL in NAFLD patients
Su et al., Medicine (Baltimore), 2015 Aug. [71]	300 cancer-free cases	SNP rs1800625 of RAGE gene increases risk of HCC but has an inverse association with HCC progression	5 common polymorphisms of RAGE gene were tested
	265 HCC patients		
D'Adamo et al., Free Radic Res, 2013 Mar. [72]	Obese prepubertal children with NAFLD	Vitamin E supplementation increased the levels of esRAGE after 6-month treatment	Vitamin E could potentially be a treatment in obese children with NAFLD
	(i) 24 children received vitamin E supplementation	Vitamin E supplementation decreased oxidative stress and improved cardiometabolic alterations	
	(ii) 21 age- and sex-matched received life-style intervention		
Gaens et al., Journal of Hepatology, 2012 Mar. [73]	74 obese individuals	CML and CML upregulated RAGE-dependent inflammatory response associated with NAFLD	In vitro model of steatosis used
			CML accumulation associated with increased gene expression of proinflammatory cytokines
D'Adamo et al., Antioxidants and Redox signaling, 2011 Mar. [74]	140 prepubertal obese children	esRAGE and sRAGE levels were significantly lower in obese prepubertal children affected by liver steatosis compared with the group without liver steatosis	Definition of liver steatosis by ultrasound instead of the gold-standard invasive liver biopsy
	71 boys and 69 girls aged between 6 and 10 years		
Kimura et al., J Gastroenterol, 2010 Jul. [75]	43 patients with dyslipidemia and biopsy-proven NASH	Atorvastatin reduces the serum levels of AGEs	AGEs are useful biomarkers for the attenuation of NASH
Yilmaz et al., Clin Biochem, 2009 Jun. [76]	40 NASH	Patients with NASH (definite and borderline) had lower plasma levels of sRAGE	
	8 borderline NASH		
	9 simple fatty liver		
	14 healthy controls		
Hyogo et al., J Gastroenterol Hepatol, 2007 Jul. [77]	66 NASH	The serum glyceraldehyde-derived AGE level may be a useful biomarker for NASH	Constant increase in glyceraldehyde-derived AGEs could potentially contribute to the pathogenesis of NASH
	10 simple steatosis		
	30 controls		

## Abbreviations

ADAM10: A disintegrin and metalloproteinase domain-containing protein 10
AGE: Advanced glycation end‐products
AGE-R: AGE receptor complexes
A*β*: Amyloid-*β* peptide
BMI: Body mass index
CAP: Controlled attenuation parameter
C1q: Complement component 1q
CML: *Nε*-(carboxymethyl)lysine
cRAGE: Cleaved RAGE
ER: Endoplasmic reticulum
esRAGE: Endogenous soluble RAGE
FAS: Fatty acid synthase
FCHL: Familial combined hyperlipidemia
HCC: Hepatocellular carcinoma
HMGB1: High mobility group box 1
LPA: Lysophosphatidic acid
LPS: Lipopolysaccharides
MAC-1: Macrophage adhesion ligand-1
MMP: Matrix metalloproteinase
MS: Metabolic syndrome
MUFA: Monounsaturated fatty acids
NAFLD: Nonalcoholic fatty liver disease
NASH: Nonalcoholic steatohepatitis
NBNC-HCC: Non-B or non-C hepatocellular carcinoma
RAGE: Receptor for advanced glycation end-products
SNP: Single-nucleotide polymorphisms
sRAGE: Soluble RAGE
TG: Triglyceride.
